# Novel *ARX* mutation identified in infantile spasm syndrome patient

**DOI:** 10.1038/s41439-020-0094-2

**Published:** 2020-03-31

**Authors:** Yohei Takeshita, Tatsuyuki Ohto, Takashi Enokizono, Mai Tanaka, Hisato Suzuki, Hiroko Fukushima, Tomoko Uehara, Toshiki Takenouchi, Kenjiro Kosaki, Hidetoshi Takada

**Affiliations:** 1 Department of Pediatrics, Ibaraki Seinan Medical Center Hospital, Sakai-machi, Japan; 2 Department of Pediatrics, University of Tsukuba Hospital, Tsukuba, Japan; 3 Department of Child Health, Faculty of Medicine, University of Tsukuba, Tsukuba, Japan; 4 Center for Medical Genetics, Keio University School of Medicine, Tokyo, Japan

**Keywords:** Disease genetics, Development

## Abstract

We report a 7-year-old boy with infantile spasms caused by a novel mutation in the *Aristaless-related homeobox* (*ARX*) gene. He showed infantile spasms and hypsarrhythmia on electroencephalogram from early infancy. Brain MRI did not reveal severe malformation of the brain except mild hypoplasia of the corpus callosum. Two-fold adrenocorticotropic hormone (ACTH) therapy failed to control the seizures, and ketogenic diet therapy and multi-antiepileptic drug therapy were required as he showed intractable daily tonic-clonic seizures. Exome sequencing identified a hemizygous mutation in the *ARX* gene, NG_008281.1(ARX_v001):c.1448 + 1 G > A, chrX: 25025227 C > T (GRCh37). To our knowledge, this mutation has not been reported previously.

X-linked infantile spasm syndrome-1 (ISSX1), also known as early infantile epileptic encephalopathy-1 (EIEE1; MIM 308350, X-linked recessive), is characterized clinically by infantile spasms, hypsarrhythmia and severe psychomotor retardation. ISSX1 is caused by missense mutations or expansion mutations in the polyalanine tract in the *Aristaless-related homeobox* (*ARX*) gene, as well as intellectual disabilities and epilepsy without severe brain malformation, including X-linked intellectual disability with or without dystonia and Ohtahara syndrome^1^. Here, we present a 7-year-old boy with infantile spasm syndrome caused by a novel mutation of the *ARX* gene, as confirmed by exome sequencing.

The 7-year-old patient was the second child born to healthy nonconsanguineous Japanese parents. The family history was unremarkable. The boy was born at 39 weeks gestation without asphyxia. The birth weight was 2844 g.

Tonic spasms involving flexion of the upper limbs were observed from 1 month of age, and he was referred to our hospital with involuntary movement at 3 months of age. He showed no visual response or head control, and the general muscle tone was poor. Brain MRI did not reveal severe malformation of the brain except mild hypoplasia of the corpus callosum. Various blood tests, including amino acid analysis, lactate and pyruvate, cerebrospinal fluid test, urine organic acid analysis, and ophthalmologic examination, were within the normal limits. Karyotype analysis showed a normal male karyotype of 46,XY. As the electroencephalogram revealed hypsarrhythmia, it was diagnosed as infantile spasm syndrome. Adrenocorticotropic hormone (ACTH) injection therapy (0.015 mg/kg/day) was started at 4 months of age. Seizure and hypsarrhythmia, however, did not disappear after 2 weeks of therapy, and the dose of ACTH was increased to 0.025 mg/kg/day from the third week. Four weeks after starting therapy, it was stopped due to elevation of liver enzymes (aspartate aminotransferase (AST) 162 IU/L, alanine aminotransferase (ALT) 215 IU/L, gamma-glutamyl transferase (GGT) 123 IU/L). High-dose phenobarbital therapy in combination with potassium bromide, zonisamide, and benzodiazepines was ineffective. The second ACTH therapy was performed from 10 months of age, but the convulsions continued, and the patient started to have a daily tonic-clonic seizure. Ketogenic diet therapy and multi-antiepileptic drug therapy relieved the seizures, which remained intractable. He showed profound intellectual disability and spastic tetraplegia and was tube-fed via gastrostoma. An exome analysis was performed at the age of 7.

Prior to an exome analysis, informed consent from the parents and approval from the University of Tsukuba Hospital review board, based on the tenets of the Declaration of Helsinki, were obtained for the molecular studies. Written consent was obtained from the patient’s parents. Genomic DNA was extracted from peripheral blood leukocytes of the patient and his parents. An exome analysis of the trio was performed as described previously^2^. Briefly, all the exons were captured using the SureSelect All Exon V6 Kit (Agilent Technologies, Santa Clara, CA); then, exome analyses were performed using the HiSeq2500 platform (Illumina, San Diego, CA).

Exome sequencing showed a hemizygous mutation in the *ARX* gene, NG_008281.1(ARX_v001):c.1448 + 1 G > A, chrX: 25025227 C > T (GRCh37), which was not identified in his parents, resulting in truncation of the ARX protein without an aristaless domain (Figs. 1 and 2). To our knowledge, this mutation has never been reported previously, and no healthy person with this mutation was found in the Genome Aggregation Database (gnomAD), the Human Genetic Variation Database (HGVD) or the Tohoku Medical Megabank Organization (ToMMo) database.

**Fig. 1:**
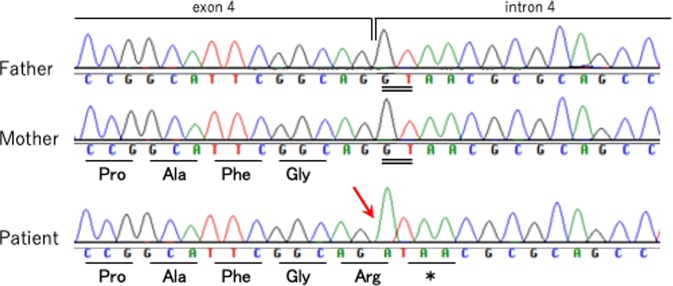
Sanger sequencing results of the *ARX* gene in the patient and his parents and predicted amino acid sequences. The patient had an *ARX*:c.1448 + 1 G > A de novo mutation (red arrow), which caused the termination of the ARX protein. The double line indicates the splice donor site at the 5′ end of intron 4.

**Fig. 2:**
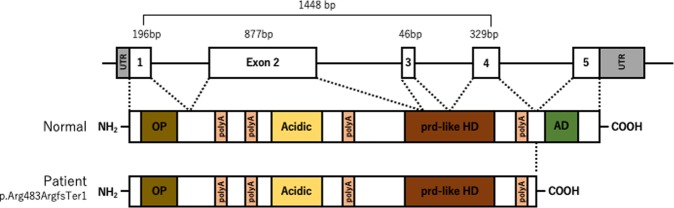
Schematic representation of the ARX gene and predicted protein. Genomic structure of the *ARX* gene and approximate protein domains and important functional regions of ARX, including the octapeptide domain (OP), polyalanine tract (polyA), acidic domain (Acidic), prd-like homeodomain (prd-like HD), and aristaless domain (AD). The number above the exons indicates the sizes in base pairs (bp). The predicted ARX protein in the patient is truncated and has no aristaless domain. Modified from Gage et al.^14^ and Ohira et al.^6^.

The Aristaless-related homeobox (ARX) protein, the vertebrate homolog of Drosophila Aristaless, belongs to the Aristaless-related subset of the paired (Prd) class of homeodomain proteins^3^. It is located in Xp21.3 and has a central homeodomain (HD) and a conserved C-terminal aristaless domain (AD). It also has three polyalanine (polyA) tracts N-terminal to the HD and a fourth polyA tract between the HD and AD^4,5^. In situ hybridization of ARX in human fetal brain sections at various developmental stages showed the highest expression in neuronal precursors in the germinal matrix of the ganglionic eminence and in the ventricular zone of the telencephalon. Expression was also observed in the hippocampus, cingulate, subventricular zone, cortical plate, caudate nucleus, and putamen. These findings suggest that ARX plays crucial roles in the differentiation and maintenance of specific neuronal cell types in the human brain^6^.

Mutations in *ARX* cause a broad variety of neurologic symptoms, ranging from severe brain malformation to structurally normal brains having intellectual disabilities and epilepsy^7,8^. In addition, there is a reasonably close phenotype−genotype correlation in the *ARX* gene. In a review of 29 males with *ARX* mutations, Kato et al. found that those with premature termination or nonsense mutations had brain malformation syndromes, including LISX2 and Proud syndrome, whereas those with missense and expansion of the polyA tract had epileptic encephalopathy or intellectual disability without brain malformations^1,9^.

Our patient’s mutation (*ARX*:c.1448 + 1 G > A) is thought to cause failure to splice intron 4, as the splice donor site at the 5′ end of intron 4 would not be recognized. Predicted pre-messenger RNA should have information of intron 4 with stop codon (TAA) near the 3′ end of exon 4, resulting in the truncation of ARX protein between the fourth polyA tract and the AD (Fig. 2). A mutation in the splice acceptor site at the 3′ end of intron 4, *ARX*:c.1449-1 G > C, has been reported in a boy with early-onset developmental and epileptic encephalopathy, and this mutation was also predicted to abolish the splice acceptor site, retaining intron 4 and leading to a premature termination codon immediately after exon 4^10^. As exon 5 is the last exon of the *ARX* gene, it would be predicted to escape nonsense-mediated mRNA decay (NMD), and analysis of cDNA from the lymphoblastoid cells of this patient confirmed retention of intron 4 and loss of detectable expression of *ARX* mRNA across exon 4 to exon 5. Although mRNA analysis was not performed in our case, there is a possibility that our mutation also causes the loss of *ARX* mRNA expression after exon 4, escaping NMD, because it results in the same intron 4 splicing failure. The predicted ARX protein in our case has no AD, and the phenotype and genotype were similar to those previously reported as intellectual disability and infantile onset developmental and epileptic encephalopathies with deficit of AD^10,11^, suggesting that our patient’s mutation should be pathological.

Genotype−phenotype correlation in animal models with *ARX* mutation has been reported as compatible with that in humans. In *Arx*-deficient male mice, aberrant migration and differentiation of interneurons containing gamma-aminobutyric acid (GABAergic interneurons) in the ganglionic eminence and neocortex resembled some of the clinical features of X-linked lissencephaly with abnormal genitalia (XLAG) in humans^12^. On the other hand, a mouse model expressing the human ARX c.428_451dup24 duplication mutation, the most frequent human mutation in polyA tract 2, presented with hyperactivity, enhanced stereotypes and alerted contextual fear memory^13^. These animal models are relevant for the investigation of the pathophysiological effects of these mutations and for therapeutic approaches because of their similarities with human patients.

In conclusion, we identified a novel *ARX* mutation (c.1448 + 1 G > A) in a Japanese boy who presented with infantile spasm syndrome without severe brain malformation.

## Data Availability

The relevant data from this Data Report are hosted at the Human Genome Variation Database at 10.6084/m9.figshare.hgv.2826.
